# Decreased intravenous glucose intake safely prevents hyperglycemia in postsurgical children

**DOI:** 10.1186/cc9820

**Published:** 2011-03-11

**Authors:** CT De Betue, SC Verbruggen, H Schierbeek, S Chacko, JB Van Goudoever, KF Joosten

**Affiliations:** 1 Erasmus MC, Rotterdam, the Netherlands; 2 Baylor College of Medicine, Houston, TX, USA; 3 Academic Medical Centre University of Amsterdam/VU Medical Centre, Amsterdam, the Netherlands

## Introduction

Critical illness induced hyperglycemia in critically ill children can be treated with intensive insulin therapy, but hypoglycaemia is a potential serious side effect. We have investigated whether decreasing intravenous glucose intake, as an alternative method, improves plasma glucose levels without affecting glucose production and protein balance in postsurgical children.

## Methods

Eight children (age 9.8 ± 1.9 months, weight 9.5 ± 1.1 kg) admitted to the pediatric ICU after surgical correction for nonsyndromal craniosynostosis were studied in a randomized blinded cross-over setting to receive standard glucose (SG, 5.0 mg/kg/minute) or low glucose (LG, 2.5 mg/kg/minute). A 10-hour stable isotope tracer protocol was conducted 6 hours after surgery to study glucose and protein metabolism.

## Results

During SG, hyperglycemia (>110 mg/dl) was present, while LG resulted in normoglycemia (LG 105 ± 10 vs. SG 133 ± 30 mg/dl; *P *= 0.02), but not in hypoglycemia. Endogenous glucose production increased during LG (LG 2.6 ± 1.5 vs. SG 1.1 ± 1.4 mg/kg/minute; *P *= 0.05). Whole body protein balance was slightly negative in both groups and was not affected by glucose intake.

## Conclusions

Standard glucose intake in postsurgical children induced hyperglycemia. Decreasing the intake by one-half of current standards resulted in normoglycemic levels, with increased endogenous glucose production. Patients were in a slight catabolic state and decreasing glucose intake did not deteriorate this. Decreasing glucose intake is a safe method to prevent hyperglycemia in critically ill postsurgical children.

